# Estimating Access to Drinking Water Supply, Sanitation, and Hygiene Facilities in Wolaita Sodo Town, Southern Ethiopia, in Reference to National Coverage

**DOI:** 10.1155/2016/8141658

**Published:** 2016-11-29

**Authors:** Amha Admasie, Ashenafi Debebe

**Affiliations:** School of Public Health, Wolaita Sodo University, Wolaita Sodo, Ethiopia

## Abstract

*Introduction*. The coverage of sanitation and access to safe drinking water in Ethiopia especially in Wolaita Sodo town are not well studied. Therefore, the main objective of this study was estimating access to drinking water supply, sanitation, and hygiene facilities in Wolaita Sodo town, southern Ethiopia, in reference to national coverage.* Methods*. A community based cross-sectional study design method was employed in the study in 588 households of Wolaita Sodo town inhabitants. Face-to-face interview to household owners, in-depth interview to key informants, reviewing secondary data, and observational check lists were used to collect data. Districts were selected using simple random sampling techniques, while systematic random sampling technique was applied to select households. Data was analyzed using Epi Info version 3.5.4 and SPSS version 16 statistical software. Bivariate and multivariable logistic regression analysis were carried out.* Results*. The community has access to improved water supply which was estimated to be 67.9%. The main water sources of the town were tap water within the yard, which was estimated to be 44.7%, and tap water in the community was 40.0% followed by private protected well which was 14.5%. Ninety-one percent of the households had at least one type of latrine in their homes. The most common type of latrine available to households was pit latrine with superstructure which was estimated to be 75.9% followed by a pit without superstructure, 21.3%, and more than half of the respondents had hand washing facilities in their compound. Occupational status, educational status, and training on water, sanitation, and hygiene related topics were significantly associated with use of improved water source, improved sanitation, and hygiene facilities.* Conclusion*. In order to address the demand of the town, additional water, sanitation, and hygiene programs are required.

## 1. Background

The health and wellbeing of population are directly affected by the coverage of water supply and sanitation [1–3]. The impact of poor environmental conditions on the transmission of communicable disease is well established. The disease burden from water, sanitation, and hygiene is estimated to be 4.0% of all deaths and 5.7% of the total disease burden (in DALYs) occurs worldwide [4]. High incidences of childhood diarrhea, helminthiasis, trachoma, and high mortality rates are associated with poor sanitation and water supply facilities [5, 6]. Excreta contain a wide variety of human pathogens and removal of these pathogens from the immediate environment has a dramatic impact on health of the community [4, 7].

Access to safe water, adequate sanitation, and hygiene facilities can mitigate a person's risk of diarrheal disease [8, 9]. The provision of safe and adequate water supply, proper disposal of human excreta and refuse, the control of the safety of food, vegetables, and beverages from disease causing organisms or their poisonous products, and the control of flies, lice, mosquitoes, and so forth are man's first line of defense against disease [10].

A high incidence of enteric diseases associated with poor sanitation is characteristic of the disease picture in many developing countries of the world [11]. The best ways of combating these diseases from cost-benefit and cost-effectiveness points of view are the provision of safe drinking water, the practice of food hygiene, and the sanitary disposal of excreta [11–13].

A research conducted to review available national and state/territory survey data on water supply and sanitation in remote Indigenous Australian communities and to discuss the findings in terms of priorities for health and infrastructure development inferred that many communities do not have a reliable water supply and experience frequent and prolonged breakdown in sewerage systems [14]. Items of basic household infrastructure regarded as essential for household hygiene are missing or not functional in many community-owned dwellings. For example, in about one-third of houses bathroom taps and toilet drainage required major repairs [14].

Basic water supply, sanitation, and hygiene facilities are showing an increasing pattern in Ethiopia from time to time [15], but data on the access to drinking water supply, sanitation, and hygiene facilities are limited in Ethiopia [16–18] specifically in Wolaita Sodo town; therefore the objective of this study was to estimate access to drinking water supply, sanitation, and hygiene facilities in Wolaita Sodo town, Wolaita Zone, southern Ethiopia.

## 2. Methods

### 2.1. Study Site

The study was conducted in Wolaita Sodo town in southern Ethiopia. The town is located at a distance of 330 km south of Addis Ababa (a capital of Ethiopia). It has three subcities and 11 districts (lower level administration). The town has 100,755 populations and 15,850 households.

### 2.2. Study Design

A community based cross-sectional study was conducted in May to July 2014 using a pretested semistructured questioner supplemented by qualitative methods obtained by in-depth interview of the local water supply official and water quality technician and health extension workers of the town.

### 2.3. Sampling

The study area has three subcities, a total of 11 districts. Two districts were taken randomly from each subcity to form a total of 6 districts. The minimum sample size was determined by single population proportion formula by considering local assumptions. Based on these assumptions, five hundred eighty-eight (588) households were determined and these households were selected across each of the six districts based on the number of households proportion to each district. Houses were selected using systematic random sampling techniques based on the roster list of each household. For the qualitative data collection, three in-depth interviews (one for water supply office, one for water quality technician, and one for town health extension worker) were conducted. For the in-depth interview, it was moderated by an experienced person who had made many interviews and moderations previously and the in-depth interview was recorded with tape recorder; in addition hand written notes were taken during the interview by note taker and further transcribed and translated into English.

### 2.4. Data Collection Tools

The data was collected using semistructured questionnaires, in-depth interview, reviewing secondary data, and observational checklist [19]. The questionnaire was designed to obtain information on sociodemographic characteristics of household owners, sources of water supply, and availability and accessibility of water container, amount of water consumption, and sanitation and hygiene information and disease condition information. The main focus areas addressed during the in-depth interview were how sanitation is maintained in the community, how people practice hygiene and sanitation, how water quality is assessed, and which factors affect the water, sanitation, and hygiene practice in the community.

### 2.5. Operational Definition

An unimproved water source is water from a dam or pool or stagnant water from a river, stream, or rainwater tank. Improved water sources are water piped into the residence, from a human-powered drill or from a water tower. Unimproved (poor) sanitation status houses have no latrine or toilet facility. Households with improved (good) sanitation status houses have a pour-flush latrine or ventilated improved pit latrine. Poor hygiene practice includes having no hand washing and bathing facilities or detergents in the house or washing hands with water but no soap or other detergents. Good hygiene practices include the use of hand washing and bathing facilities, with the availability of soap and other detergents in the house [19].

### 2.6. Data Quality Assurance

To assure the quality of the data in the study, the English version questionnaire was translated to Amharic (local language) and back translated to English by translators who were blind to the original questionnaire. Data collectors and supervisors were trained and a regular supervision and follow-up were made by principal investigators. Pretest was done in nonselected districts. The collected data was reviewed and checked for completeness and consistence by the supervisors and principal investigators each day. The data was checked, coded, and entered into computer and cleaned before analysis.

### 2.7. Variables

Variables were classified as two main ones, namely, demographic variables such as age, sex, education status, occupation status, ethnicity, religion, and family size, and environmental health variables such as water supply, hygiene, and sanitation.

### 2.8. Data Processing and Analysis

For quantitative data, Epi Infor version 3.5.4 and SPSS version 16 were used and then some of the responses were randomly selected and checked for errors during data entry. Then a descriptive frequency was used for checking of outliers. Data was cleaned accordingly and further analysis was done. Bivariate and multivariate logistic regression analyses were carried out. Odds ratio, 95% confidence interval, and p values were determined for each variable.

### 2.9. Ethical Consideration

Ethical permission to undertake the study was obtained from Wolaita Sodo University Research and Community Service Directorate. Official letter of cooperation was given to Wolaita Sodo City Administration. Informed consent to participate in the study was obtained before conducting the interview. For this a one-page consent letter was attached to the cover page of each questionnaire and it will explain to study participants that participation is voluntary and confidential and private information was protected. The right of the respondent to withdraw from the interview or not to participate was respected. Identification of an informant was possible only through specific identification numbers.

## 3. Results

### 3.1. Demographic Characteristics of Respondents

A total of 588 households were approached and all were participated with a response rate of 100%. The data was collected from all the three subcities in the town. The majority (422) (71.8%) of respondents were females. Regarding the age of respondents, 305 (51.9%) were >31 years of age and 279 (47.4%) were between 16 and 30 years of age. Regarding educational status of the respondents, 144 (24.5%) were unable to read and write followed by grade 1–6 level which was estimated to be 163 (27.7%). Concerning their occupation of respondents, 208 (35.4%) of the participants had business related occupations. Finally, 236 (40.1%) of the respondents had a family size >5 families (Table 1).

### 3.2. Water Supply Condition

The main drinking water sources in the study area were estimated to be 399 (67.9%) and 189 (32.1%) from improved water source and unimproved water source, respectively. But specifically, the main water sources of respondents, tap water within the yard, and tap water in the community were 263 (38.0%) and 235 (33.9%), respectively.

Most of the participants (465) (48.70%) stored their drinking water using a container made up of plastic materials (the capacity ranges from 20 to 5000 liters; it depends on the socioeconomic status of the household) and barrel users were 274 (28.70%). The most common type of cleansing material to clean the water storage container was water with soap which was estimated to be 495 (78.10%) and followed by water without any detergent only (87) (13.70%).

Water sources reliability was only perennial (148) (26%), and the remainders (427) (74%) were intermittent (i.e., water was not available throughout the year, especially during dry seasons from February to June). In almost all households (99%), water storage container has a covering material and 268 (45.6%) of the respondents clean their water storage container twice in a week while 177 (30.1%) of them did it once in a week.

Most of the town depends upon piped water supply, in supplement with river and private well water sources to meet the demand. However, the share of the river water and private well water sources increases during the warmest season of the year (from February to June).

Even though 529 (90.0%) of the households heard about household water treatment methods, only 378 (71.5%) of the participants ever used either of the household water treatment methods. The most dominant type of water treatment methods used was disinfection (using chemicals like chlorine, aqua-tabs, and other locally manufactured water disinfectants or chlorine stock preparations), which was estimated to be 333 (61.0%) (Table 2).

### 3.3. Sanitation and Hygiene Status

Ninety-one percent of the households have at least one type of latrine. The commonest type of private latrine, which was available in the community, was a pit latrine with superstructure and estimated to be 407 (75.9%), followed by a pit latrine without superstructure, 114 (21.3%). The coverage of household toilet facility was higher as compared to the national coverage (which was 84% of urban town that has access to improved toilet facility).

Almost all types of latrine (530) (98.9%) were found functional; moreover 425 (79.3%) of the latrines were clean during the time of visit. Three hundred nine (57.6%) of the latrines have attached to a hand washing facility in or around the latrine. Five hundred thirty-two (90.5%) of the families have a trend of washing their hand just after they visited a latrine.

The very fascinating result from the community was concerning the hand washing. Hand washing facilities were almost available to all households. All families washed their hands after a toilet visit. Due to such good practice, the status of diarrheal disease was declining in the community from previous experience.

Training on hygiene and sanitation practice has been given to 539 (91.7%) of the families. Those trainings were provided by different stakeholders; among training providers, about 493 (91.5%) and 26 (4.8%) of the families were trained by health extension workers and Woreda health office health professional, respectively.

Those households that had no private latrine estimated to be 44 (84.6%) used a communal latrine, and none of the respondents reported using a public latrine. Mostly the latrine was cleaned by girls, estimated to be 371 (69.2%) and followed by mothers (212) (39.6%). The families had a habit of hand washing after toilet and 453 (85.2%) of the families used water and soap followed by water only (113) (21.2%) (Table 3).

### 3.4. Water and Sanitation Related Disease Condition

Diarrheal disease was one of the problems faced in the study area. There were 42 (7.1%) of the respondents that had complaints of any type of diarrheal disease in the last 2 weeks preceding the date of interview. Among these complaints, 19 (45.2%) of cases were children under five. Among the diarrheal diseases complaints, only 28 (4.8%) of the diseases were confirmed as waterborne diseases.

### 3.5. Factors Associated with Water Sources

As per the definition of WHO/UNICEF [19] of unimproved water source and improved water source put in the operational definitions, there were few factors that determine the status of the water sources in that community. Occupation of the head of the households, water source reliability status, and frequency of fetching water from any type of water sources and the availability of information on water treatment were associated with the quality of water source within the community (Table 4).

### 3.6. Factors Associated with Sanitation and Hygiene

As per the definition of WHO/UNICEF [19] of unimproved water source and improved waters put in the operational definitions, educational status of the head of the households, their occupation, use of soap after toilet visit, presence of hand washing facility, and access to wash training were associated with the sanitation and hygiene of the community (Table 5).

### 3.7. Results In-Depth Interview

The discussion from key informants had generated supplementary evidence during the interview in the community and sometimes had paradox responses to the actual problem in the community.

### 3.8. Water Supply

Even though majority of the community consumes tap water, the other group of people used river water, unprotected hand dug well, and surface water as well. Consuming unsafe water is considered to be a risk factor to health by the community, but sometimes there is no other option not to use this poor quality water. The responses from the key informants were a paradox; for example, one key informant replied “*Generally, water supply system of the town was running in good pattern, which the water generating, storage system, and water distribution system was working in a very coordinating way. The authority tests water quality for physical, chemical and microbiological parameter at regular interval of time.*” The other key informant expressed the situation as “*many people's request that to have tap water in their yard and community, but this is very difficult to address all at a time. The only option we are doing is to increase their awareness how to treat water at home level and providing chemical disinfectant*.* Most peoples in the outskirt of the town never accessed to safe and adequate water supply, even the tap water supply is very intermittent or irregular supply.*”

### 3.9. Sanitation

“*Recently, the sanitation system of the town was improving but still much open defecation is observing everywhere*” is the reply by one of the key informants. Most people used to urinate in the ditches and street flood canals instead of looking for toilets in their home. “*The available public latrines were insufficient. Few were already collapsed and still looking for new construction. Adequate health information had been provided to a home-to-home, but sometimes people might not afford to construct latrine by their own.*” Regarding solid waste disposal, it is very improved according to the key informant that says “*We mobilize and privatize the solid waste collection and disposal system to small scale enterprises. Each household are paying for the service. But still solid waste is a problem in the community, wastes are disposing openly and in to the nearby rivers and drainage ditches.*”

### 3.10. Hand Washing

Hand washing was a good culture to all, but people were not consistent in using hand washing tradition as a tool to reduce diarrheal disease. Studies indicated that hand washing reduces diarrhea diseases significantly [20, 21]. “*To the community, the common practice of hand washing is before eating food. The reason for poor hand washing habit after a toilet visit could be lack of water, lack of hand washing facilities, and poor awareness*” as responded by the key informant. “*We demonstrate how hand washing facility as it can be made using locally available materiasl [sic], but they did not want to refill water frequently. Only few peoples use soap for hand washing facilities during our visit to house-to-house*”. Even though it is a low cost sanitation improving tool, there is still significant gap in utilization of hand washing facilities according to our observation during the visit time.

## 4. Discussion

The study revealed that the overall water supply coverage of the town was reasonably inadequate in all subcities of the town, but the physical access of improved water source to the community was 67.9% which was lower than the EDHS 2005 report, 93.7% [17], and EDHS 2011 report, 94.5% [18], respectively. The possible explanation of the lower improved water supply status could be a problem of distribution infrastructure and supply system [15] and additionally the town is rapidly growing and the infrastructure and the rate of urbanization were incomparable. According to the study, among improved water source users, only 72% of the community used tap water, which was very low as compared to the national (Ethiopia) tap water users, which was 90% [17] but according to Joint Monitoring Report, Ethiopia's water supply coverage has improved from year to year but the figures reported by the government were different from nongovernment organizations [22–24]. Above sixty percent of the respondents had used chemical disinfection for homemade water treatment. The study showed that there was a higher water treatment practice in the household level as compared to the Ethiopia Demographic Health Survey Report, 2005, at which only 8% of the households treat water at household prior to drinking [17].

The study showed that there were factors such as type of occupation of the households, water source availability, and training taken regarding the water safety significantly associated with use of improved water sources. Occupationally, government employees were 2.47 times more likely to use improved water sources than other occupations (OR = 2.47, 95% CI: 1.08–5.63). Those individuals who had not taken training on household water treatment methods were 0.26 less likely to use improved water sources than who had taken the training (OR = 0.26, 95% CI; 0.12–0.55). This was evidenced with the study done in Burkina Faso that hygiene promotion reduces the childhood diarrhea [25].

Ninety-one percent of the households have at least one type of latrine which was higher than the EDHS 2011 report, 68% [18]. Despite the progress seen in Ethiopia, 28% practice open defecation [26]. The most common type of private latrine available to households was pit latrine with superstructure which was estimated to be 407 (75.9%) followed by a pit without superstructure (114) (21.3%). The coverage of household toilet facility was higher as compared to the national coverage (at which 84% of urban town has access to improved toilet facility) [27] and better than Benin, which was 8.7% [28]. Educational status and occupation of the individuals were significantly associated with the presence of latrine facilities in their home. Those individuals who had educational status of grades 1–6 were 4.02 more likely to have latrine facilities than those whose educational status was higher education and above (OR = 4.02, 95% CI: 1.28–12.56). Even though the variable is significantly associated, no evidence supports such an association elsewhere in the study.

Those individuals who were businessmen in their occupation were 3.08 more likely to have latrine facilities in their house than individuals who have other occupations (OR: 3.08, 95% CI: 1.26–7.50). This could be explained due to the economic factor that businessmen are more able to afford costs incurred to construct latrine facilities than others.

Almost all types of latrine (530) (98.9%) within the households were functional; moreover 425 (79.3%) of the latrines were clean during the time of visit. Three hundred nine (57.6%) of the latrines have attached to a hand washing facility in or around the latrine. Five hundred thirty-two (90.5%) of the family have a trend of washing their hand just after they visit a latrine. The result revealed that it was better coverage as compared to similar studies in the northern Ethiopia [29].

## 5. Conclusion

The water coverage was too low to address the water demand of the rapidly urbanizing town. Even though the drinking water supply coverage was in line with the national figure, still there was a drinking water supply problem observed in the town. There was a significant gap in distribution of water supply infrastructure in the town. Even though the community used tap water as a primary source of water for any type of domestic purposes, they also used private well as an alternate source for most of the community. It was a good practice to use different cleansing material for cleaning purpose of water containers. Though most latrines were pit latrine with superstructure, almost all the households in the town have a latrine, and more than half of them attached to hand washing facilities in or around the latrine. Very few households complain of diarrheal diseases within the last week, but almost nearly half of the affected segments of the community were children. Additional capital investments required to address the demand of the town population with water supply are required. The local water authority has to complete already begun projects within very short period of time. It was not possible for the government to address the demand of water supply; therefore public-private participation must be encouraged to maintain the water, sanitation, and hygiene sector of the town and new ways of financing for the sector should also be explored. The urban health extension programs have brought significant differences in water, sanitation, and hygiene promotion in the country and this should be encouraged to be continued.

## Figures and Tables

**Table 1:** Sociodemographic characteristics of households of Wolaita Sodo town, 2013.

Variables	Responses	Number	Percent
Sex of the respondents	Male	166	28.2
	Female	422	71.8

Age of respondent (in years)?	<15 years	4	0.7
	16–30 years	279	47.4
	>31 years	305	51.9

Marital status of head of the household	Single	28	4.8
	Married	477	81.1
	Divorced	23	3.9
	Widowed	60	10.2

Educational status of head of the household	Unable to read & write	144	24.5
	Grades 1–6	163	27.7
	Grades 10–12	142	24.1
	Higher education	139	23.6

Occupation of head of the household	Business related	208	35.4
	Government employee	163	27.7
	Daily laborer	97	16.5
	Unemployed	34	5.8
	Other	86	14.6

Number of family members in the household	<5 families	177	30.1
	5 families	175	29.8
	>5 families	236	40.1

**Table 2:** Drinking water supply status of households of Wolaita Sodo town, 2013.

Variables^*∗*^	Respondents response	Responses		Percent of cases
		Number	Percent	
Drinking water source (*n* = 588)	Improved	399	67.90%	67.90%
	Unimproved	189	32.10%	32.10%

The main source of drinking water (*n* = 588)^*∗*^	Private protected well	85	12.30%	14.50%
	Private unprotected well	19	2.70%	3.20%
	Tap water within yard	263	38.00%	44.70%
	Tap water in community	235	33.90%	40.00%
	River water	58	8.40%	9.90%
	Others	33	4.80%	5.60%

*Total*		*693*	*100%*	*117.90%*

Drinking water storage materials (*n* = 588)^*∗*^	Plastic material	465	48.70%	79.10%
	Bucket	180	18.90%	30.60%
	Barrel	274	28.70%	46.60%
	Clay pot	33	3.50%	5.60%
	Others	2	0.20%	0.30%

*Total*		*954*	*100.00%*	*162.20%*

Type of cleansing materials for clean water containers? (*n* = 586)^*∗*^	Water only	87	13.70%	14.80%
	Water with soup	495	78.10%	84.50%
	Water with ash and leaves	52	8.20%	8.90%

*Total*		*634*	*100.00%*	*108.20%*

Have you ever heard about water treatment methods? (*n* = 588)^*∗*^	Yes	529	90%	90%
	No	59	10%	10%

If you have ever used household water treatment methods, which methods do you use? (*n* = 378)^*∗*^	Storage (sedimentation)	90	16.50%	23.80%
	Filtration (cloth)	23	4.20%	6.10%
	Sand filtration	4	0.70%	1.10%
	Disinfection (chemical)	333	61.00%	88.10%
	Boiling	91	16.70%	24.10%
	Others	5	0.90%	1.30%

*Total*		*546*	*100.00%*	*144.40%*

How reliable are the water sources? (*n* = 575)	Perennial	148	26%	26%
	Intermittent	427	74%	74%

Do all the water storage containers have covers? (*n* = 588)	Yes	584	99%	99%
	No	4	0.7%	0.7%

^*∗*^Multiple responses type of questions.

**Table 3:** Sanitation and hygiene facilities of households of Wolaita Sodo town, 2013.

Characteristics	Variables	Responses		Percent of cases
		Number	Percent	
Does your household have latrine? (*n* = 588)	Yes	536	91.2%	91.2%
	No	52	8.8%	8.8%

If the household has private latrine, what type of latrine do you have? (*n* = 536)	Pit without super structure	114	21.3%	21.3%
	Pit with super structure	407	75.9%	75.9%
	VIPL	11	2.1%	2.1%
	Flash toilet	4	0.7%	0.7%

Is there a hand washing facility in or around the latrine? (*n* = 536)	Yes	309	57.6%	57.6%
	No	227	42.4%	42.4%

Does the family have washing hands after using toilet? (*n* = 588)	Yes	532	90.5%	90.5%
	No	56	9.5%	9.5%

If the family have a habit of hand washing after toilet, what do they use to wash their hands? (*n* = 532)^**∗**^	Water only	113	18.60%	21.20%
	Water and soap	453	74.60%	85.20%
	Water & ash	38	6.30%	7.10%
	Others	3	0.50%	0.60%

*Total*		*607*	*100.00%*	*114%*

If your family members ever received training on hygiene and sanitation practices, what sort of training was it? (*n* = 539)^**∗**^	Water handling	419	30.00%	77.70%
	Latrine construction	485	34.80%	90.00%
	On personal hygiene	484	34.70%	89.80%
	Other specifications	7	0.50%	1.30%

*Total*		*1395*	*100%*	*258.80%*

Has any of the family members ever received training on hygiene and sanitation practices? (*n* = 588)	Yes	539	91.70%	91.70%
	No	49	8.30%	8.30%

^*∗*^Multiple responses type of questions.

**Table 4:** Determinant factors to use different water supply sources, in Wolaita Sodo town, 2013.

Questions	Responses	Improved water source	Unimproved water source	COR (95% CI)	AOR (95% CI)
Sex of the respondents	Male	88	78	0.40 (0.27–0.58)	0.53 (0.22–1.22)
	Female	311	111	1.00	1.00
Occupation of the respondents	Business work	144	64	1.54 (0.92–2.60)	1.79 (0.85–3.79)
	Gov. employee	124	39	2.18 (1.24–3.82)	*2.47 (1.08–5.63)*
	Daily labor	56	41	0.93 (0.51–1.69)	1.09 (0.47–2.48)
	Unemployed	24	10	1.64 (0.70–3.86)	2.45 (0.76–7.95)
	Others	51	35	1.00	1.00
Water source reliability	Perennial	37	111	0.07 (0.04–0.11)	*0.16 (0.08–0.31)*
	Seasonal	353	74	1.00	1.00
Water fetching frequency for domestic purposes	Every day	98	130	0.18 (0.07–0.45)	*0.17 (0.04–0.81)*
	Twice a week	276	53	1.24 (0.48–3.19)	042 (0.09–1.97)
	Once a week	25	6	1.00	1.00
Clean water storage containers	Daily	70	62	1.97 (0.55–7.07)	4.35 (0.89–21.14)
	Twice a week	187	81	4.04 (1.15–14.18)	3.92 (0.84–18.15)
	Once a week	138	39	6.19 (1.72–22.24)	4.35 (0.93–20.25)
	Once a month	4	7	1.00	1.00
Information on household water treatment methods	No	23	36	0.26 (0.14–0.49)	*0.26 (0.12–0.55)*
	Yes	376	153	1	1.00
Hand washing facility around latrine	No	134	93	0.59 (0.67–0.76)	0.78 (0.48–1.26)
	Yes	226	83	1	1.00

**Table 5:** Contributing factors to have latrine facilities in Wolaita Sodo town, 2013.

Questions	Responses	Latrine	No latrine	COR (95% CI)	AOR (95% CI)
Sex of the respondent	Male	159	7	22.71 (10.65–48.42)	2.48 (0.97–6.32)
	Female	377	45	1.00	1.00
Educational status of the head of the household	Unable to read & write	133	11	12.091 (6.53–22.36)	*8.58 (2.30–31.86)*
	Grades 1–6	152	11	13.818 (7.49–25.48)	*4.02 (1.28–12.56)*
	Grades 7–12	123	19	6.474 (3.99–10.49)	1.48 (0.53–4.09)
	Higher education	128	11	1.00	1.00
Occupation of head of the household	Business work	194	14	13.857 (8.05–23.83)	*3.08 (1.26–7.53)*
	Gov. employee	146	17	8.588 (5.19–14.19)	1.28(0.48–3.33)
	Daily labor	95	2	47.5 (11.70–192.70)	*21.71 (2.68–175.2)*
	Unemployed	30	4	7.5 (2.64–21.28)	1.31 (0.35–4.84)
	Others	71	15	1.00	1.00
Number of family members	<5 families	161	16	10.062 (6.02–16.82)	1.1 (0.49–2.47)
	5 families	158	17	9.294 (5.63–15.32)	0.76 (0.35–1.65)
	>5 families	217	19	1.00	1.00
Water source status	Unimproved	176	13	13.538 (7.70–23.78)	1.22 (0.51–2.92)
	Improved	360	39	1.00	1.00
Training on wash practices	Yes	47	2	23.5 (5.70–96.74)	2.40 (0.55–14.75)
	No	489	50	1.00	1.00
